# Outcome of Combined Hepatocellular and Cholangiocarcinoma of the Liver

**DOI:** 10.1155/2010/917356

**Published:** 2010-09-02

**Authors:** Jue Wang, Fenwei Wang, Anne Kessinger

**Affiliations:** ^1^Section of Oncology-Hematology, Department of Internal Medicine, University of Nebraska Medical Center, Omaha, NE 68198-7680, USA; ^2^Department of Internal Medicine, Creighton University, Omaha, NE 68131, USA

## Abstract

*Background*. The objective of this study was to examine the epidemiology, natural history, and prognostic factors of combined hepatocellular and cholangiocarcinoma (cHCC-CC) using population-based registry. *Methods*. The Surveillance, Epidemiology, and End Results Program database (1973–2004) was used to identify cases of cHCC-CC. Multivariable logistic regression was used to evaluate factors associated with cancer-directed surgery (CDS). The influence of CDS on cancer specific survival was evaluated using Kaplan-Meier curves and Cox proportional hazards modeling. *Results*. A total of 380 cases of cHCC-CC were identified, which account for approximately 0.87% of primary liver tumors. Of all patients, 69.8% of patients had regional or distant stage; 65.6% of patients had poorly or undifferentiated histology. Only 44.9% of patients with localized disease, received CDS. By logistic regression analysis, being widowed, advanced stage, and earlier diagnosis year were associated with lower rate of utilization of CDS. In multivariate analysis, tumor stage, receipt of CDS, and recent year of diagnosis were found to be significant predictors for cancer-specific survival. *Conclusions*. Patients with localized cHCC-CC who are selected for CDS were strongly associated with improved survival. However, many patients with localized tumors did not receive potentially curative cancer-directed surgery. Further study is warranted to address the barriers to the delivery of appropriate care to these patients.

## 1. Introduction

Combined hepatocellular and cholangiocarcinoma (cHCC-CC) is an uncommon subtype of primary liver cancer [1, 2]. The disease was first described in 1949 by Allen and Lisa and has been defined as the intimate intermingling of both a HCC component and CC component (2). Two histopathological classification schemes have been proposed (1, 2). Allen and Lisa [1] described three groups, type A with HCC and CC present at different sites within the same liver, and type B with HCC and CC present at adjacent sites and mingle with continued growth, and type C with HCC and CC are combined in the same tumor. Goodman et al. [2] categorized cHCC-CC into three types: (i) collision type, (ii) transitional type, and (iii) fibrolamellar type. Kim et al. [3] and Zhang et al. [4] proposed that cHCC-CC is a distinct type of primary liver carcinoma, which is morphologically and phenotypically intermediate between HCC and CC and may be derived from hepatic progenitor cells with the bipotential to differentiate into both hepatocytic and cholangiocytic lineages. 

Because of the rarity of cHCC-CC, previously published literature has been based on case series and anecdotal experiences [3–14], with largest case series include thirty cases. The majority case series so far were reported from Asia. Most of these reports were histopathological studies, the demographic features and clinical behavior of this disease remain ill-defined, and the outcomes of these patients are varied in a number of studies. For example, the reported results of surgical resection from single institution have been inconsistent [10, 15–19]. In this study, we take advantage of the vast amount of data collected by the SEER Program to examine the largest series of cHCC-CC reported to date. We examine the incidence, natural history, utilization of cancer-directed surgery (CDS) as well as prognostic factors that might affect the survival for cHCC-CC.

## 2. Methods

### 2.1. Data Source

In 1973, the SEER registry program was established to identify all new cancer cases diagnosed within 7 geographic areas [20]. Since 1973, the SEER Program has expanded several times to improve representative sampling of minority groups as well as to increase the total sampling of cases to allow for greater precision. The original SEER 9 registries included Atlanta, Connecticut, Detroit, Hawaii, Iowa, New Mexico, San Francisco-Oakland, Seattle-Puget Sound, and Utah. By 1975, SEER included 9 geographic regions, 5 states (Connecticut, Hawaii, Iowa, New Mexico, and Utah) and 4 metropolitan areas (San Francisco-Oakland, Seattle-Puget Sound, Detroit, and Atlanta). In 1992, four additional registries were added to form the SEER 13 registries, which included the SEER 9 registries, plus Los Angeles, San Jose-Monterey, rural Georgia, and the Alaska Native Tumor Registry. More recently, in 2000, data from greater California, Kentucky, Louisiana, and New Jersey were added to the SEER 13 Program to form the SEER 17 registries. SEER 9, 13, and 17 registries cover approximately 9.5%, 13.8%, and 26.2% of the total U.S. population, respectively [20]. Data for this study were obtained from SEER*Stat public-use data files available from the National Cancer Institute. For incidence analyses, registry data were linked to total U.S. population data from 1969 to 2003.

### 2.2. Study Population

The cases of cHCC-CC were extracted from the SEER on the basis of anatomic site (ICD-O-2 codes C22.0–22.1) and histological type (ICD-O code 8180) for those patients first diagnosed and/or treated between January 1973 and December 2004. A total of 42,654 patients with hepatobilliary neoplasms were identified in the SEER 17 registries, among them, 380 patients were found with cHCC-CC. Cases identified at the time of autopsy or by death certificate only (24 cases) were excluded from survival analyses.

### 2.3. Variables

Patients' social demographic characteristics (i.e., age, race/ethnicity, and marital status) and tumor grade and stage at the time of diagnosis, were determined from the SEER database. 

 Because there is no AJCC (The American Joint Committee on Cancer) staging system for cHCC-CC, general summary stage was used [21]. This system classifies patients as having local, regional (extension into adjacent tissues or nodal involvement), or distant disease. The World Health Organization's standard grading system was used with four separate categories (well, moderately well, poorly differentiated, and undifferentiated). For analysis purpose, they were grouped into low grade (well, moderately well differentiated) and high grade (poorly differentiated, and undifferentiated). 

In SEER database, any treatment that is given to modify, control, remove or destroy primary or metastatic cancer tissue is considered to be cancer-directed surgery (CDS). CDS was defined in this study as surgical resection (hepatectomy), transplantation, local regional therapy (such as radiation frequency ablation, chemoembolazation, and embolization), and unknown surgery, based on values for site-specific surgery and surgery of the primary site codes within the database [21]. Since only few patients received local regional therapy, we grouped these patients together with patients underwent unknown surgery together as “other surgery”.

### 2.4. Statistical Analysis

By using linked population files, age-adjusted incidence rates and their 95% confidence intervals (CIs) were calculated for cHCC-CC for all patients, for men and women separately, and for each of the 3 broad categories of race (whites, blacks, and other) and in three time periods. 

 Discrete data are reported as frequencies and compared by chi-square tests. Continuous data are reported as mean ± SD and compared by Student's *t*-test. Multivariate logistic regression analyses were used to determine the factors associated with receipt of cancer-directed surgery. Survival duration was measured by the Kaplan-Meier method and compared by the log rank test. Multivariable Cox proportional hazards model was used to identify independent predictors of long-term cancer specific death. 

SEER*Stat 6.2.4 (Surveillance Research Program, National Cancer Institute) was used for incidence analyses [20]. All other statistical calculations were performed by SPSS 12.0 (Apache Software Foundation 2000). Survival durations calculated by SPSS. Comparative differences were considered statistically significant when the *P* value was <.05.

## 3. Results

### 3.1. Frequency and Incidence

Between 1973 and 2004, a total of 380 patients with cHCC-CC were identified from 42,654 patients with hepatobilliary neoplasms, which consisted of 0.87% of all patients with hepatobillary cancers. A constant age-adjusted incidence of 0.03 per 100,000 was observed in these three time periods. Detailed incidence data by time period, gender, and race are included in Table 1.

### 3.2. Patient and Tumor Characteristics

Table 2 includes the patient and tumor characteristics of study cohort. Of the 380 patients with cHCC-CC identified in the SEER database, there was male predominance with a male-to-female ratio of 1.8 : 1. The mean age of patients with cHCC-CC was 64 ± 12 years (median age 65 years, with a range of 19 to 98 years). The majority of patients (76.6%) were white. African American accounted for 43 cases (11.03%). Other ethnicities accounted for 46 cases (12.11%). 

Staging information were not available for 55 (14.5%) patients. Of the remaining 325 patients, 98 (25.8%) were classified as localized stage; 97 (25.5%) were classified as regional stage; and 132 (34.2%) were classified as distant stage. 82 of 125 (65.6%) patients whose histology information available had poorly or undifferentiated histology.

### 3.3. Treatment

Cancer-directed surgery was performed for 79 (20%) patients, among them, 20 patients underwent liver transplantation, 40 patients underwent partial hepatectomy, 5 patients underwent local surgery (4 patients received radiofrequency ablation (RFA) and 1 patient received percutaneous ethanol injection (PEI)), and the rest of 14 patients underwent unknown surgery. A total of 301 (79.1%) patients were treated nonsurgically. Radiation therapy was performed in a total 22 (5.8%) of patients, in 4 (1.1%) of 22 patients, radiation was used as an adjuvant to surgery (Table 2). 

In a logistic regression analysis, marital status, tumor stage, and year of diagnosis were identified as independent predictors of receiving CDS. The patients who were widowed, patients with advanced stage and those who were diagnosed before 1989, were less likely receiving CDS (Table 3). In a separate analysis restricted to patients with local and regional disease, the above factors remained independent predictors of CDS.

### 3.4. Survival Analysis

The mean followup duration of the entire cohort was 8.4 months. A total of 341 of 380 (89.7%) patients died during the followup period. 

 For survival analysis, we excluded the cases that were identified at autopsy or on the basis of death certificates only. A total of 356 patients were included in cancer-specific survival analysis. The median overall survival for all cases was 4 months (95% CI 3–5). 

**Figure 1:**
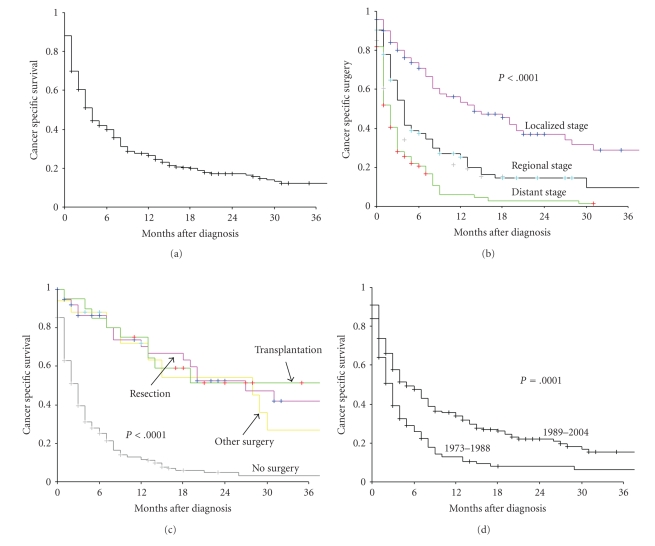
**(**a) Cancer-specific survival for overall patients with combined hepatocellular and cholangiocarcinoma of the liver (cHCC-CC). (b) Cancer-specific survival rate of patients according to SEER stage. *P* < .0001 for Localized versus Regional versus Distant stage. (c) Cancer-specific survival rate of patients with localized disease according to treatment. *P* < .0001 for transplantation versus resection versus other surgery* versus no surgery. (d) Cancer-specific survival rate of patients according to year of diagnosis. *Other surgery: local surgery plus unknown surgery.

Figure 1 presents the cancer specific survival rates according to patient and tumor characteristics. Cancer-specific survival rates for entire cohort at 1-, 3-, and 5-year were 26.5%, 12.4%, and 9.2%, respectively, (Figure 1(a)). The 1-, 3-, and 5-year cancer-specific survival rates for patients with local and regional stage tumor were 56.3%, 29.0%, 22.1%, 25.3%; 9.6%, and 4.8%, respectively, and for patients with distant stage tumor were 6.1%, 1.5%, and 0%, respectively, (Figure 1(b)). There was a significant difference in survival between patients who underwent CDS (transplantation, resection or other surgery) versus those did not (*P* < .0001) (Figure 1(c)). The outcomes of patients with cHCC-CC were significantly improved for patients who were diagnosed in later years (1989–2004) compared to those in earlier years (1973–1988) (Figure 1(d)). 

Table 4 presents the result of multivariate survival analyses using Cox proportional hazard model. After adjusting for the demographic, clinical, and treatment-related factors, tumor stage, receiving CDS, and year of diagnosis were identified as independent predictors of cancer-specific survival. Compared to patients with localized disease, patients with regional and distant disease had 1.62 and 2.5 fold increased risk of dying, respectively. The most important predictor of outcome was CDS. Patients who receive cancer directed surgery had significant decrease in the risk of dying than those patients who did not. (Transplantation versus no CDS, HR = 0.25; hepatectomy versus no CDS, HR = 0.26; other surgery (local treatment and unknown surgery) versus no CDS, HR = 0.28).

## 4. Discussion

The reported frequency of combined tumors in series of primary hepatic malignancies varies widely, from 1.0–4.7% [1, 2, 11, 15]. In this study, cHCC-CC accounted for approximately 0.87% of primary liver tumors during study period, which is lower than what was reported in single institution studies [13]. “Referral bias” may contribute to the observed higher incidence rates in the single institution studies, which usually were from tertiary hospitals and referral centers. Patients with rare histology subtypes are more likely to visit referral centers for second opinion; pathologists in tertiary centers usually have more opportunity seeing these cases and having expertise to identify these cases, compare with community counterparts. Therefore, the incidence rate found in a population-based study, which include wide spectrum of hospitals, are more likely to reflect the true incidence of this rare tumor.

Similar with previous studies, most of patients with cHCC-CC presented with high histological grade tumors and advanced stage disease at the time of presentation (Table 2). Our findings, along with others [22], supports the notion that cHCC-CC represents a distinct, aggressive subtype of liver cancer [4–11]. These findings of subtype-related differences in liver cancer have significant implications: better therapy of liver cancer is more likely to be achieved by investigating each subtype of liver cancer separately rather than grouping them all together. As our understanding of the genetic basis for cancer grows, it is likely that liver cancer will be subdivided into ever finer categories. 

 Surgery is the only treatment offering the possibility of a cure. The main treatment goal should be complete excision with negative margins and limited impact on liver function. In our cohort, only 20% of all patients received CDS. This likely reflects their advanced stage on presentation. The majority of patients unfortunately were not candidates for potential curative CDS. In addition less than half of the patients with localized disease underwent CDS (Table 2) raises a concern whether cancer-directed surgery was underused in this population. The findings of disparity of CDS utilization in cHCC-CC are similar with findings in studies in HCC [23, 24], efforts should be made to reduce these disparities. The patients diagnosed between 1989 and 2004 were almost three times more likely to receive CDS, in comparison to those diagnosed between 1973 and 1988. This encouraging trend of increasing utilization CDS is in line with the recent advances in radiologic diagnosis [25, 26] and a shift towards an earlier stage at diagnosis [22, 27], and advances in liver surgery technique [14, 28, 29]. 

 Although the role of liver transplantation in the management of HCC or cholangiocarcinoma is well defined [30, 31], data about the role of liver transplantation in the management of cHCC-CC are lacking [32]. Our study represented the largest series of surgical management of patients with cHCC-CC. At present, liver transplantation and resection are the only potential curative therapy for cHCC-CC, therefore, surgery should be considered in patients when complete resection is possible. Because, liver resection carries considerable operative risk; poor performance status and comorbidities may have precluded some patients from resection.

 Currently, there is no literature available addressing impact local regional treatment options on survival specifically on cHCC-CC. Transarterial chemoembolisation (TACE) and percutaneous treatments such as percutaneous ethanol injection (PEI) and radiofrequency ablation (RFA) are widely used treatments for unresectable HCC and postresection recurrence [33, 34]. However, many cHCC-CCs are less vascular and much more fibrotic than HCC, and thus are less likely to respond to TACE or PEI [35]. Our findings suggest RFA is useful in selected patients; however, the small numbers of the patients underwent this therapy and the short duration of followup, makes any conclusions unreliable, and more research is required to confirm this finding.

 Consistent with single institution studies, the prognosis of this disease was poor [15, 17, 18], the median cancer specific survival for the patients with distant disease was 2 months (range 0–31 months). We did, however, observe a significant improvement in the outcome of patients with cHCC-CC over time (Figure 1(d)). Since there was no effective chemotherapy for liver cancer during the study period [36], the better outcomes likely reflect the advance in surgical techniques, better perioperative management and supportive care [14, 28, 29].

 Our findings should be interpreted within the limitations of the study. Although we adjusted for differences in demographic and tumor factors, residual confounding might still be present. Unlike single-institution studies, the accuracy of staging and pathologic diagnosis within a national registry may vary widely across the institutions. In addition, SEER data did not allow us to examine surgical volume, and patient's comorbidities, all of which may influence survival in cancer patients. However, the use of cancer specific survival rather than overall survival in our study has modified the limitation to some degree. 

Strengths of this study include the populations-based design and the large sample size. Having large sample size is of particular importance for analysis of rare tumors such as cHCC-CC, where it is nearly impossible for a single institution to collect enough cases to make meaningful analysis regarding important prognostic factors.

## 5. Conclusions

The findings of this study confirmed that CDS was associated with statistically significant increase in cancer-specific survival. However, fewer than 50% patients with localized disease received CDS. Further study is warranted to explore and address the potential barriers to the delivery of appropriate care to these patients.

## Figures and Tables

**Table 1:** Age-adjusted incidence rate of combined hepatocellular and cholangiocarcinoma of the liver (cHCC-CC) per 100,000 populations.

Age-adjusted incidence rate per 100,000 (95% CI)	
SEER 9 (1973–1991)	

Overall	0.03 (0.02–0.04)
Men	0.05 (0.03–0.07)
Women	0.02 (0.01–0.03)
White	0.03 (0.02–0.04)
Black	0.02 (0.02–0.04)
Other	0.07 (0.03–0.12)

SEER 13 (1992–1999)	

Overall	0.03 (0.02-0.03)
Men	0.04 (0.01–0.03)
Women	0.02 (0.01-0.02)
White	0.03 (0.02-0.03)
Black	0.03 (0.00–0.03)
American Indian	0.00 (0.00–0.03)
Asian	0.03 (0.00–0.03)

SEER 17 (2000–2003)	

Overall	0.03 (0.02-0.03)
Men	0.04 (0.01–0.03)
Women	0.02 (0.01-0.02)
White	0.03 (0.02-0.03)
Black	0.03 (0.01–0.07)
American Indian	0.00 (0.00–0.15)
Asian	0.03 (0.00–0.06)

SEER: surveillance, epidemiology, and end results.

CI: confidence interval.

**Table 2:** Characteristics of 380 patients with combined hepatocellular and cholangiocarcinoma of the liver (cHCC-CC) diagnosed between January 1973 and December 2004.

Characteristics	*N* (%)	CDS	No. CDS	*P* value
Gender				
Male	245 (64.5%)	49	196	.61
Female	135 (35.5%)	30	105	

Race				
Black	43 (11.3%)	5	38	.09
White	291 (76.6%)	60	231	
Others	46 (12.1%)	14	32	

Civil Status				
Married	222 (58.4%)	59	163	.01
Divorced/Separated	37 (9.7%)	7	30	
Single	52 (13.7%)	6	45	
Widowed	57 (15%)	7	51	
Unknown	12 (3.2%)	0	12	

Grade				
Well differentiated	9 (2.4%)	1	8	<.00001
Moderately differentiated	34 (8.9%)	17	17	
Poorly differentiated	70 (18.4%)	21	49	
Undifferentiated	12 (3.2%)	2	10	
Unknown	255 (67.1%)	38	217	

SEER Stage				
Localized	98 (25.8%)	44	54	<.0001
Regional	97 (25.5%)	22	75	
Distant	130 (34.2%)	11	119	
Unknown	55 (14.5%)	2	53	

Year of diagnosis				
1973–1988	154 (40.5%)	14	140	<.0001
1989–2004	226 (59.5%)	65	161	

Cancer Directed Surgery				
Yes	79 (20.8%)	79	0	<.0001
No	301 (79.2%)	0	301	

Radiation Therapy				
Yes	22 (5.8%)	4	18	.55
No	354 (93.2%)	75	279	
Unknown	4 (1.0%)	0	4	

SEER: surveillance, epidemiology, and end results.

**Table 3:** Multivariate Logistic Regression Analysis of Factors Associated with Cancer Directed Surgery.

Characteristics	Group	OR	(95% CI)	*P* value
Age		1.00	(0.97–1.03)	.95

Gender	Female	1.00		
	Male	0.57	(0.29–1.12)	.10

Race	White	1.00		
	Black	0.33	(0.11–1.02)	.053
	Other	0.99	(0.43–2.32)	.99

SEER Stage	Localized	1.00		
	Regional	0.32	(0.16–0.64)	.001
	Distant	0.12	(0.05–0.27)	<.0001
	Unstaged	0.05	(0.01–0.21)	<.0001

Grade	Low grade	1.00		
	High grade	0.63	(0.24–1.63)	.34
	Unknown	0.42	(0.18–0.99)	.05

Civil Status	Married	1.00		
	Widowed	0.22	(0.07–0.66)	.007
	Divorced/Separated	0.71	(0.24–2.08)	.53

Diagnosis Year	1973–1988	1.00		
	1989–2004	2.90	(1.38–6.12)	.005

Radiation	No	1.00		
	Yes	2.60	(0.76–8.92)	.13

OR: odds ratio; CI: confidence interval; low grade: well differentiated/moderately differentiated; high grade: poorly differentiated/undifferentiated; SEER: surveillance, epidemiology, and end results.

**Table 4:** Cox proportional multivariate analysis of factors associated with cancer-specific mortality.

Characteristics	Group	HR	(95% CI)	*P* Value
Age		1.00	(0.99–1.01)	.56

Gender	Male	1.00		
	Female	1.27	(0.93–1.71)	.14

Race	White	1.00		
	Black	1.41	(0.93–2.13)	.10
	Others	1.36	(0.91–2.03)	.13

SEER Stage	Localized	1.00		
	Regional	1.59	(1.10–2.33)	.018
	Distant	2.50	(1.70–3.61)	<.0001

Grade	Low	1.00		
	High	1.45	(0.90–2.32)	.13
	Unknown	1.02	(0.66–1.57)	.94

Civil Status	Married	1.00		
	Widowed	1.21	(0.81–1.83)	.36
	Divorced /Separated	0.96	(0.59–1.54)	.85
	Single	1.01	(0.68–1.49)	.96
	Unknown	0.61	(0.31–1.24)	.17

Diagnosis Year	1973–1988	1.00		
	1989–2004	0.81	(0.60–1.09)	.16

Radiation	No	1.00		
	Yes	0.82	(0.50–1.38)	.47

Cancer Directed Surgery	No	1.00		
	Transplantation	0.25	(0.13–0.50)	<.0001
	Resection	0.26	(0.15–0.44)	<.0001
	Other Surgery	0.28	(0.15–0.53)	<.0001

HR: hazard ratio; CI: confidence interval; low grade: well differentiated/moderately differentiated; high grade: poorly differentiated/undifferentiated; other surgery: local surgery plus unknown surgery; SEER: surveillance, epidemiology, and end results.
